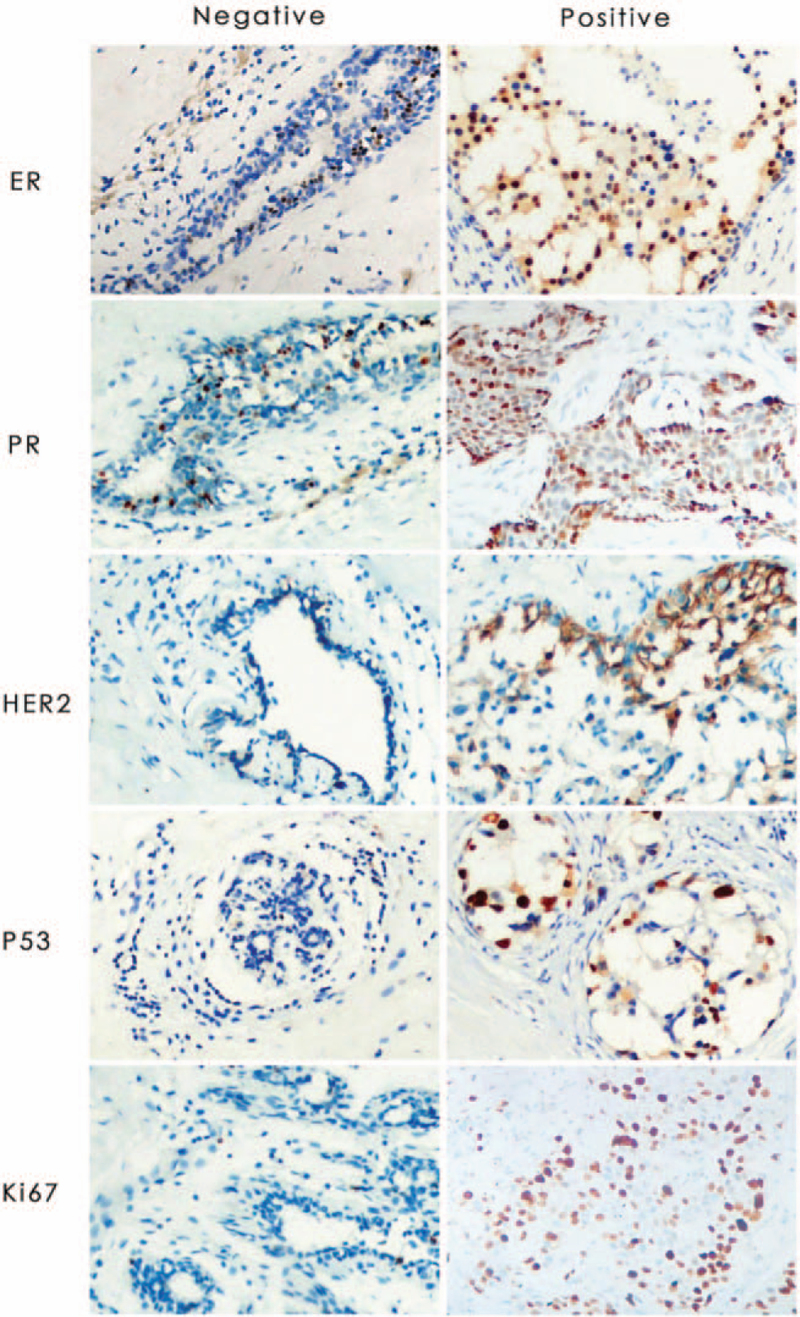# Neoadjuvant chemotherapy reduces the expression rates of ER, PR, HER2, Ki67, and P53 of invasive ductal carcinoma: Erratum

**DOI:** 10.1097/MD.0000000000028714

**Published:** 2022-01-28

In the article, “Neoadjuvant chemotherapy reduces the expression rates of ER, PR, HER2, Ki67, and P53 of invasive ductal carcinoma”,^[1]^ which appears in Volume 98, Issue 2 of *Medicine*, Figure 1 has been replaced with the below figure.

**Figure:**